# Superspreading of SARS-CoV-2 at a choir rehearsal in Finland—A computational fluid dynamics view on aerosol transmission and patient interviews

**DOI:** 10.1371/journal.pone.0302250

**Published:** 2024-09-12

**Authors:** Anna Tuhkuri Matvejeff, Alpo Laitinen, Marko Korhonen, Lotta-Maria Oksanen, Ahmed Geneid, Enni Sanmark, Ville Vuorinen

**Affiliations:** 1 Department of Otorhinolaryngology and Phoniatrics - Head and Neck Surgery, Helsinki University Hospital, University of Helsinki, Helsinki, Finland; 2 Department of Mechanical Engineering, Aalto University, Espoo, Finland; Kore University of Enna: Universita degli Studi di Enna ’Kore’, ITALY

## Abstract

**Introduction:**

COVID-19 pandemic has highlighted the role of aerosol transmission and the importance of superspreading events. We analyzed a choir rehearsal in November 2020, where all participants, except one who had recently earlier recovered from COVID-19, were infected. We explore the risk factors for severe disease in this event and model the aerosol dispersion in the rehearsal room.

**Materials and methods:**

Characteristics of participants were collected by interviews and supplemented with patient records. A computational simulation of aerosol distribution in the rehearsal room and the efficacy of potential safety measures was conducted using the Large-Eddy Simulation approach. Infection risk was studied by analyzing quanta emission and exposure with the Wells-Riley equation.

**Results:**

The simulation showed that airborne transmission likely explains this mass contagion event. Every singer was exposed to the virus in only 5 min from the beginning of the rehearsal, and maximum concentration levels were reached at 20 min the concentration levels started to approach a steady state after 20 min. Although concentration differences existed in the room, risk levels near (1 m) and far (5 m) from the aerosol source were similar for certain singers. Modeling indicated infection risk levels of 70–100% after one hour; the risk would have been considerably reduced by wearing high-filtration respirators. Age and pre-existing comorbidities predicted more severe disease. The high incidence of illness may be partly attributed to the relatively high median age of individuals. Additionally, those admitted to the hospital had multiple underlying health conditions that predispose them to more severe disease.

**Conclusions:**

Airborne transmission and indoor space can explain this mass exposure event. High-filtration respirators could have prevented some infections. The importance of safety distances diminishes the longer the indoor event. The concept of safety distance is challenging, as our study suggests that long range airborne transmission may occur in indoor events with extended duration. We encourage informing the public, especially persons at risk, of safety measures during epidemics.

## Introduction

During the COVID-19 pandemic there has been a significant paradigm shift in the understanding of infection transmission, highlighting the crucial role of aerosol transmission for SARS-CoV-2 and other respiratory pathogens [1,2]. Airborne transmission involves the possibility of mass contagion, known as superspreading events, where a high number of infective particles concentrate in an indoor space containing multiple people, many of whom become infected. A systematic review of the first waves of SARS-CoV-2 showed that 19% of cases accounted for 80% of all transmissions globally [3].

Singing has previously been found to generate more respiratory particles than speaking or breathing, and, in general, aerosol emission has been found to increase with voice loudness [4–6]. Thus, it is not surprising that choir practices have frequently been reported as venues for superspreading outbreaks [7–9]. An epidemiologic study from Germany [10] also showed an increased risk of SARS-CoV-2 infection in professional choir singers relative to controls during the pandemic. Studies of choir outbreaks, including one of the first reported outbreaks at the Skagit Valley choir rehearsal, have all suggested airborne spread of the disease [8,9,11]. Disease severity for individual singers has been presented in each study, but the effect of a singer’s location or patient-related risk factors on the severity of the disease has not been investigated previously.

From the medical perspective, a number of risk factors increase the probability of SARS-CoV2 transmission or the severity of the disease. Age and male gender are risk factors for COVID-19 infection [12], and from the transmitters’ side, the early phase of the infection [13]. Exposure to a large number of virus particles likely increases both the transmission risk [13] and the severity of the disease [14]. Age has been shown to increase an individual’s particle emission by Bagheri et al. and Schumm et al. [5,15]. Furthermore, age is seemingly the strongest independent risk factor for severe disease [16]. Other factors elevating individual risk for severe disease include male gender, obesity, diabetes, hypertension, cardiovascular disease, chronic lung disease [17,18], immunodeficiency [19], and sleep apnea [20].

From the engineering perspective, aerosol transmission phenomena are closely related to understanding the physics of airflow transporting small particles. For example, the size and geometrics of the interior space, insufficient ventilation, exposure time, and the respiratory activity performed have been linked to increased transmission risk. Only a few computational fluid dynamics (CFD) publications using the advanced Large-Eddy Simulation (LES) approach in the SARS-CoV-2 aerosol transmission context have been reported [21–23]. Vuorinen et al. [21] examined the dispersion of aerosols in indoor spaces using LES. They noted that small aerosols remain airborne for extended periods of time and are almost immune to gravitational settling. Liu et al. [22] simulated a restaurant setting where people exposed to COVID-19 were infected using LES. They reported a clear connection between high aerosol concentrations and infection patterns. Auvinen et al. [23] evaluated how air purifiers and space dividers affect the aerosol concentrations in a restaurant using LES. Turbulent mixing was noted to be crucial to the rapid dispersion of concentration peaks. Air purifiers were deemed to enhance the turbulent mixing, while space dividers alone did not decrease the infection risk.

In this study, we aim to describe a real-life superspreading event at a senior choir rehearsal in November 2020 in Finland, where 14 of the 15 singers became infected, two of them subsequently being hospitalized [24]. The main objectives of this multidisciplinary study are as follows: First, from the engineering and physics perspective, we utilize LES to explore whether aerosol transmission could explain the infection of nearly all choir members and whether the infection risk could have been reduced. Second, from the medical perspective, we aim to record the risk factors in transmission and severity of the disease and their relative importance and disease severity in the presented superspreading event. We present the characteristics of the singers and the venue, compare the findings with existing data on risk factors for COVID-19, and present a computational view of the aerosol spread in a model setup.

## Materials and methods

### Choir rehearsal

The weekly rehearsal of a senior choir in Uusimaa, Southern Finland was held on 16 November 2020. The 90-min choir rehearsal of 15 members took place in a clubroom, where the seating was arranged in accordance with the 2-m safety distance. A similar safety distance was maintained at all times. The participants had been instructed to only attend the rehearsal in good health and without symptoms on the rehearsal day. Hand disinfectant was used before the training and there was no closer contact (i.e. hand shaking or hugging), although no masks, respirators, or other personal protective equipment were worn during or after the rehearsal.

The participants, both men and women, sang Christmas carols in unison with accordion accompaniment. After the rehearsal, some of the singers visited a nearby cafe and returned to sing karaoke in the same clubroom where the choir rehearsal had taken place. Only singers of the choir participated in the karaoke.

### Choir member characteristics

The superspreading case presented here was first reported in Finnish media. Based on the news article, we contacted the leader of the choir. At our request, the leader informed all choir members about the possibility to participate in the study. The recruitment of participants was carried out between March 11th, 2022, and December 15th, 2022.

There were no exclusion criteria in the study and all volunteer singers were recruited.

Eleven of the 15 choir members, 5 male and 6 female, volunteered to participate in the study. The structured interviews were conducted by phone in June-December 2022 (S1 Survey) to obtain general information, e.g., age and diagnoses at the time of the event, as well as disease-specific information, e.g., onset and type of symptoms. The received information was supplemented with a review of patient records that were accessed between March 21st, 2023, and April 21st, 2023 by one of the first authors (AT). An overall picture of the event was formed on the basis of interviews and photographs and written notes on the event. Other authors did not have access to information that could identify individual participants during or after data collection.

### Computational model of the room

To better understand the engineering and physics aspects of aerosol dispersion, we introduce a computational fluid dynamics (CFD) model. The CFD simulations are performed with the open-source software OpenFOAM [25]. The 3D, time-dependent airflow patterns can be obtained by solving the Navier-Stokes equations (i.e. conservation of mass, momentum, and energy for air) using the Large-Eddy Simulation (LES) approach. LES is a high-performance computing, turbulent flow simulation method offering relatively accurate information on airflow patterns and mixing processes by employing high space and time resolution. For example, at the location of the persons, we resolve flow length scales in the order of 1 cm. Here, the k-equation subgrid scale model [26] is utilized. Importantly, this LES approach also accounts for the rising buoyant heat from the attendees. In our study, we only consider the fine aerosols (e.g. dry particle diameter less than ca. 20 micrometers) that are considered to be solely responsible for the long-distance aerosol transmission in superspreading events [21]. Instead of actually solving the individual aerosol particle paths, we solve an additional convection-diffusion equation that describes how the airflow transports the concentration field. Hence, the concentration field only accounts for the smallest aerosols which are assumed to be less affected by gravitational forces and have a negligible effect on the airflow in the room.

The connection between aerosol concentration and infection risk can be understood via the ’quanta’ concept *q* ([*q*] = 1/m^3^), which is useful in understanding the infection potential of the exhaled viral aerosols. The term ’quanta’ refers to the quantity of particles in the air carrying infectious pathogens, and ’quantum’ denotes a dose of such particles required to cause infection in 63% probability. ’Quanta emission rate’ pertains to the amount of infectious particles released into the air over a specific time period, while ’quanta concentration’ denotes the density of these particles per unit volume [27]. The quanta concept is used in the classical Wells-Riley analysis for airborne transmission and the concept only considers the infectious proportion of the viral aerosol. The infection probability (*P*) can be written in terms of the mathematical expression.

$$P=1-\text{exp}\left(-p\int_{o}^{t}q\left(x,\;y,\;z,\;t^{\prime }\right)dt^{\prime }\right),$$ (1)

where *t* = exposure time, *p* = 1.2 m^3^/h is the assumed inhalation rate (see e.g. [21]), *q(x*,*y*,*z*,*t)* is the local quanta concentration level at the location of each singer at time *t*. Quanta emission rates of *q’ = 100–1000 1/h* have been reported in the literature for loud speaking and singing for the early variants of SARS-CoV-2 [27].

As a remark, we do not model the details of the exhalation air jet of the singers because of the large-scale separation between the mouth scales (e.g., 1 mm) and the room scale (e.g., 1 m). Instead, the respiratory aerosol formation is modeled as a spherical source term located around the head of the infected singer. The infected singer exhales virus in terms of quanta (*q*) at a rate *q’* ([*q’*] = 1/h). Here, we set *q’* = 1000 1/h, which may represent the pathogen release rate related to such high vocal activities as singing [27]. According to Eq 1, when a person has inhaled one quantum of the virus, the probability of infection is 63%. It should be noted that the LES CFD simulations offer a full 3D, time-dependent view of the concentration field (*q*), enabling actual local evaluation of Eq 1. More details on the computational approach along with a grid sensitivity study are provided in our earlier publication [28].

**Fig 1:**
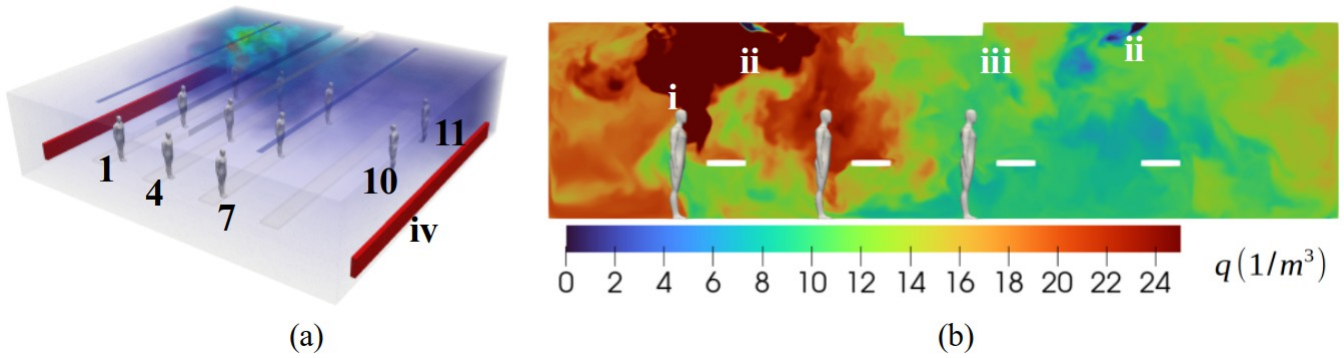
(a) Room geometry with 11 individuals showing the aerosol cloud dispersion at time t = 20 min. The dimensions of the room are 12m x 12m x 3m and the individuals are spaced 2–3 m apart from each other. (b) A cut plane of the room and aerosol concentration level are expressed as quanta ([q] = 1/m^3^). The scale is truncated at q = 25 1/m^3^. i: index person, ii: inflow air jets, iii: exhaust air, iv: radiators.

In the present study, we investigate a model room setup that is 12 m x 12 m x 3 m, presented in Fig 1. This model is a simplification of the actual setup, but it contains the important physical details considered relevant to the aerosol dispersion patterns. In the model setup, altogether 11 attendees are located in the room as heat sources. The number of attendees (11) in the computational model was chosen based on the number of participants attending the study. We assume a heat load that goes to the thermal plumes of each singer, while the heat radiation effects are neglected since they are considered significantly less important to the overall flow field structure. We assume 60 W power per person, while the two radiators at the opposing walls contribute 720 W each. Fresh air at temperature 19°C is introduced to the room at a rate of 0.42 m^3^/s, corresponding to the ACH (= air changes per hour) value of 3.5. The ACH value was obtained from the maintenance company.

### Ethical aspects

The study was conducted in accordance with the principles of the Declaration of Helsinki. Ethical permission (HUS-1186-2021) was granted by the HUS Regional Committee on Medical Research Ethics, and written informed consent was obtained from all attendees.

## Results

### Choir rehearsal

The weekly rehearsal of a senior choir in Uusimaa, Southern Finland was held on 16 November 2020. The 90-min choir rehearsal of 15 members took place in a clubroom, where the seating was arranged in accordance with the 2-m safety distance. A similar safety distance was maintained at all times. The participants had been instructed to only attend the rehearsal in good health and without symptoms on the rehearsal day. Hand disinfectant was used before the training and there was no closer contact (i.e. hand shaking or hugging), although no masks, respirators, or other personal protective equipment were worn during or after the rehearsal.

The participants, both men and women, sang Christmas carols in unison with accordion accompaniment. After the rehearsal, some of the singers visited a nearby cafe and returned to sing karaoke in the same clubroom that the choir rehearsal had taken place. Only singers of the choir participated in the karaoke.

### Characteristics of participants

The medical history and characteristics of participants are presented in Table 1. All participants were unvaccinated, as vaccinations were not yet available at this time. Of the 11 participants, 10 received a positive Reverse transcription polymerase chain reaction (qRT-PCR) test result 3–5 days after the rehearsal; one of them was asymptomatic. For the remaining nine qRT-PCR -positive participants, the symptoms started on average 3 days (range 2–4 days) after the rehearsal. Two participants (one female, one male) were hospitalized for COVID-19 infection. One of the hospitalized individuals had participated in karaoke singing and had visited the cafe. The single participant who did not contract the disease had previously recovered from COVID-19 infection eight months before the study earlier, confirmed by a positive qRT-PCR conducted at the hospital.

**Table 1:** Characteristics of participants.

	Non-infected cases	Infected cases, non-hospitalized	Infected cases, hospitalized	Total
N	1	8	2	11
Karaoke	1	5	1	7
Age, median (range)	63	70,5 (64–75)	69,5 (65–74)	69 (63–75)
Male	0	4	1	5
Female	1	4	1	6
BMI, median (range)	27	26,2 (22,4–32,3)	34,9 (30,8–39)	26,8 (22,4–39)
Smokers	0	0	0	0
Sleep apnea	0	2	1	3
Diabetes	0	0	1	1
Hypertension	0	5	2	7
Hypercholesterolemy	0	2	1	3
Heart failure with preserved ejection fraction	0	0	1	1
Atrial fibrillation	0	1	1	2

The reported symptoms included fever (6), chills (1), cough (7), sore throat (3), fatigue (4), congestion or runny nose (5), shortness of breath (3), headache (6), body or muscle ache (9), loss of sense of smell and taste (1), nausea (2), and diarrhea (3). None of the choir members succumbed to this COVID-19 infection.

### Computational fluid dynamics simulations

#### Case setup and physical observations

The singers are numbered from 1 to 11, as shown in Fig 1. The figure displays i) the hypothesized index person (singer 3) releasing viruses at the corner of the room, ii) two inflow air jets entering from the elongated gaps of width 0.2 m symmetrically from opposite ends of the room, iii) the outflow air exhaust, which consists of two symmetric air gaps of width 0.2 m in the center of the room, and iv) two radiators located at the opposing walls. The choir leader (11) and the accordionist (10) are also marked in the figure. The simulation starts from time t = 0 during which the flow field is fully developed while *q* = 0 everywhere in the room. The aerosol concentration field (*q*) is shown at time *t* = 20 min (Fig 1). The simulation time of 20 min was chosen since it corresponds to a slightly longer time than one air exchange time (approx. 17min). Additionally, the simulated concentration field after 20 min time along with the average concentration levels were considered to be almost fully developed. The turbulent character of the flow field is apparent from panel (b), which shows the scalar concentration field at a cut plane intersecting the index patient.

**Fig 2:**
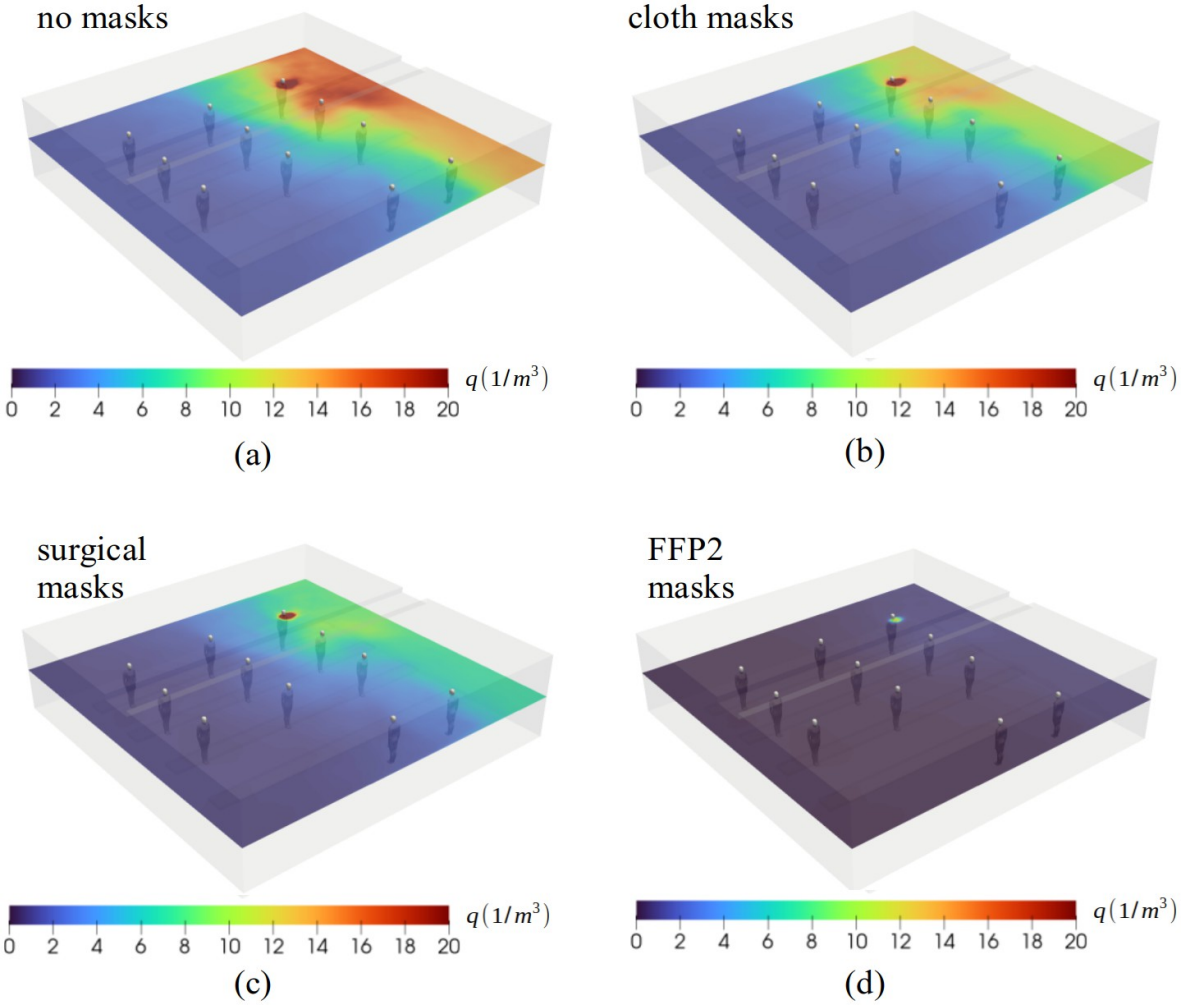
Average aerosol concentration field (q) on breathing level from time interval t = 20–25 min when the aerosol concentration levels have reached a fully developed, saturated state. The room concentration levels are directly linked to the mask filtration efficiency. The upper bound of the scale is truncated at the value q = 20 1/m^3^.

Expected aspects of airflows are noted in Fig 1; the warm rising air from the index patient lifts the viral air upwards and this air may then return to the breathing level with downward-oriented air currents. As a remark, the complexity and non-linear character of the air flow becomes clear from two observations: 1) aerosol transports from the index person to the nearest person in front of them indirectly via the ceiling of the room due to buoyancy effects and 2) although mostly concentrated around the index person, the aerosol cloud spans the entire room. For example, average concentration levels close to q = 6–12 1/m^3^, can be noted at the choir leader position (Fig 2). At the assumed inhalation rate, such concentration levels would yield a virus dose of approximately 7–14 quanta in one hour, leading to nearly 100% infection probability according to the Wells-Riley Eq (1).

#### Aerosol transmission and risk mitigation

Since the aerosol particles appear in small quantities, they do not affect the overall flow field. Hence, we can post-process and normalize our simulation data to discuss various scenarios for infection control. First, the simulations indicated that the aerosol cloud fills the entire room by time t = 15 min. Time-averaged quanta levels (1/m^3^) are calculated from the interval t = 20–25 min as shown in Fig 2. Four different scenarios are considered: no masks, cloth masks (25% filtration), surgical mask (50% filtration) and FFP2/N95 masks (95% filtration) [29]. The chosen filtration values include consideration of the mask fit, and they represent a positive, upper bound scenario.

Without masks, the minimum concentration values at t = 20 min at the breathing level are noted to be close to q = 2 1/m^3^, while the 95% filtration efficiency of FFP2/N95 respirators would decrease the respective concentration level close to q = 0.1 1/m^3^ (Fig 2). Without masks, at the assumed inhalation rate, the Wells-Riley Eq (1) would yield a minimum infection probability of approximately 70% within a 30-min exposure to q = 2 1/m^3^, while the respective probability would be approximately 0.3% if all participants are wearing FFP2/N95 respirators with decreased concentration levels (e.g., q = 0.1 1/m^3^).

**Fig 3:**
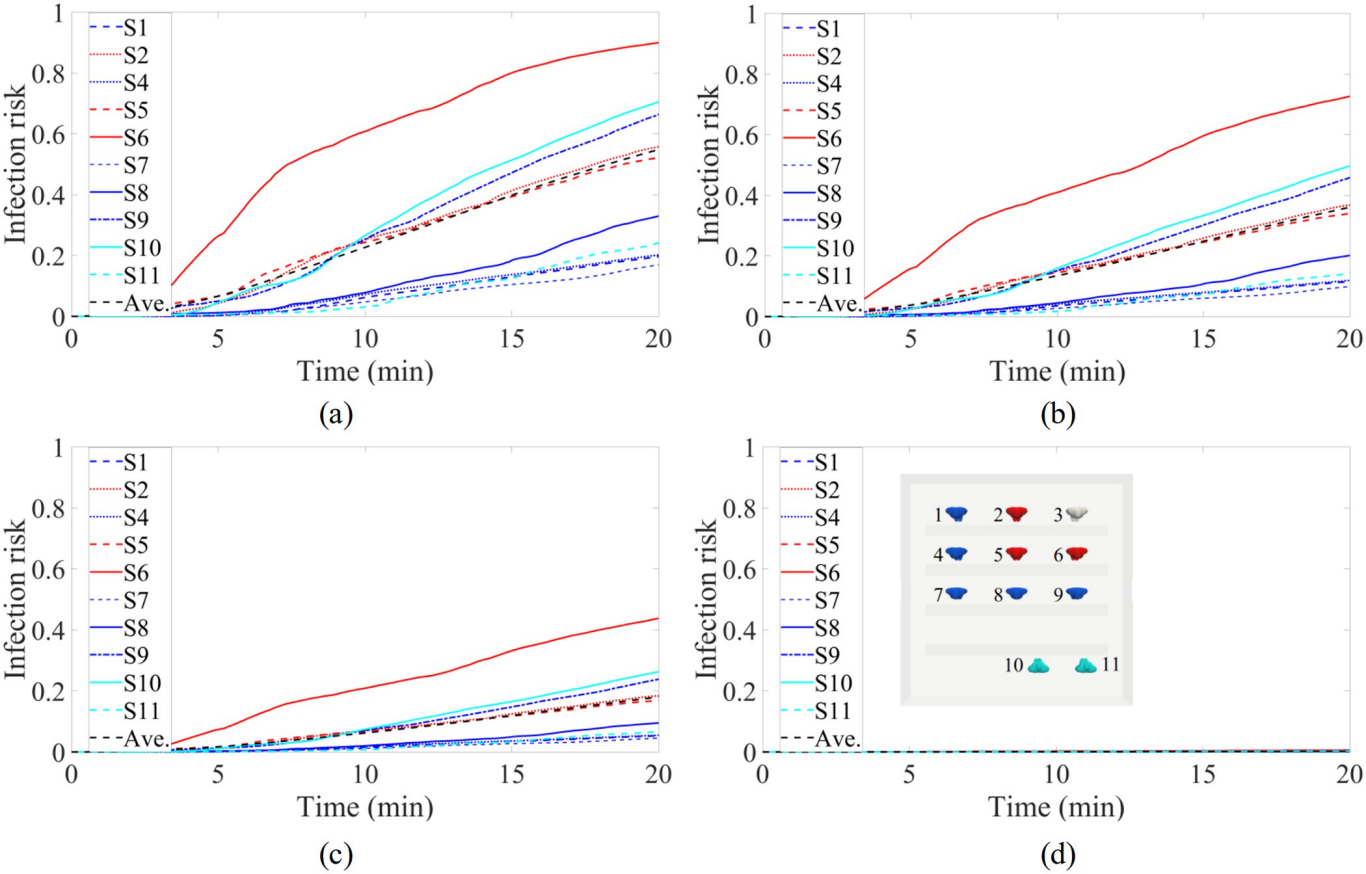
Infection risk as a function of time in four different scenarios: (a) no masks, (b) cloth masks, (c) surgical masks, and (d) FFP2/N95 masks. As shown in the inset of panel (a), singer 6 stands in front of the infected person (singer 3), while singer 2 stands next to singer 3. The colouring of the line plots is based on the distance of the singers to the index person, as shown in panel (d). A theoretical average concentration is shown as the black dashed line.

The infection risk is shown as a function of time (until t = 20 min) in four different scenarios assuming that all participants wear a similar mask (Fig 3). The infection risk based on a theoretic average concentration is also calculated. This estimate corresponds to perfect mixing conditions and would lead to almost 60% infection risk in 20 minutes for all participants without masks and less than 20% with surgical masks. The average is calculated as a weighted average concentration over the entire room. The message of the figure in the present model setup is clear; the duration of a choir practice (1–3 h) is long enough that avoiding infection with ordinary cloth masks or without any mask usage is very challenging, and most of the choir attendees would likely become infected at the assumed quanta emission rate. By contrast, the usage of high-filtration respirators (such as FFP2/N95) may offer a means of avoiding superspreading. By simple extrapolation, we note that the presently assumed quanta exhalation rate of 1000 quanta/h may maintain the infection probability of the furthermost persons (1, 4, and 7) at a range of 70–90%, while for the other participants the infection probability is very close to 100%. As a remark, the assumed quanta emission rate from singing is high, and the emission rate from breathing or talking would be much lower which would consequently lead to lower infection probability. However, the analysis of the infection risk with masks can be directly linked to scenarios without masks but with a lower quanta emission rate. For example, a 50% mask filtration level may be related to a respective decrease in quanta emission rate resulting from e.g. speaking.

## Discussion

In this paper, we investigated a mass contagion event at a senior choir rehearsal where all participants, except one who had 8 months earlier recovered from COVID-19, became infected. With computational modeling and participant interviews, we showed that 1) aerosol transmission causing the infections is likely in this mass contagion event, 2) the risk factors of participants were related to the severity of the illness, and 3) a high-quality respirator would likely have prevented at least some of the infections.

### Aerosol transmission and infection risk

As a result of the simulations, we propose that airborne transmission largely explains the superspreading event. We showed that the aerosol distribution in the room was highly non-uniform, reaching minimum concentration levels of 2 quanta/m^3^ everywhere at breathing level at a quanta emission rate of 1000 quanta/h. Such levels are sufficient to yield an infectious dose within the 1- to 2-h exposure time with a high probability. The complex physics of turbulent and buoyant airflows strongly impacts the aerosol dispersion patterns. Furthermore, the studied model predicts that the aerosol spreads relatively rapidly to the room such that every singer became exposed to the virus within 5 min from the beginning of the rehearsal, while maximum concentration levels were experienced around 20 min, being close to 1 air change time scale (approx. 17min).

### Role of safety measures

Numerical evidence from LES simulations indicates that quanta concentration levels can be similar near (1 m) and far (5 m) from the index person, which is not surprising from the fluid dynamics perspective due to the strongly buoyant character of the flow. The observation does not support the safety distance (2 m) recommendation as a sole mitigation measure indoors. The observation indicates that relatively high viral concentrations can be observed far from the source due to aerosol dispersion in indoor spaces, where ventilation capacity may not efficiently achieve dilution. Thus, the concept of a ‘safety distance’ in events like this is challenging because, if misinterpreted, it may create a false sense of safety. In fact, the results offer evidence of the poor efficacy of social distancing in preventing the spread of airborne diseases indoors as the exposure time increases. LES modeling allows for a nuanced evaluation of concentration changes occurring both spatially and temporally, uncovering discernible variations in relation to distance. This stands in contrast to conventional infection risk models, which assume perfect mixing. Consequently, this approach offers a significantly more precise insight into the evolution of infection risk as shown before [23,30]. Even though we did not investigate other indoor air quality mitigation measures, other studies have shown that improving the indoor air quality by increasing ACH, additional window ventilation, or additional air filtration devices, such as portable HEPA (High Efficiency Particulate Air) filters, may decrease the infection risk in high-risk situations [23,31]. However, In a real-life scenario, significantly increasing ACH poses challenges, including additional noise, structural vibration concerns, ventilation device limitations, energy efficiency considerations, and the need for indoor thermal comfort. Higher ventilation may also lead to medical concerns like eye and airway irritation, along with discomfort from heightened airflow perceived as a draft.

The social distancing guidelines are considered significant particularly if the exposure time is short or if the meeting occurs outdoors. Based on the present observations, the longer the exposure time, the less relevant the role of distance. The duration of normal choir practices (e.g., 1–3 h) is sufficiently long that avoiding infections with surgical cloth masks or without mask usage is very challenging, and many of the choir attendees may become infected if the quanta emission rate is high. By contrast, the usage of high-filtration respirators (such as FFP2/N95) may offer a means of avoiding superspreading. When using high-filtration respirators, even a long exposure will not increase the infection risk significantly, however we acknowledge that singing with a respirator can be considered uncomfortable. Ultimately, organizing the entire rehearsal as a remote event would eradicate the possibility of infections entirely. During future epidemics of airborne transmitted diseases, we recommend considering either one of the protective measures (respirators or remote event) in similar situations to continue the activity more safely.

### Individual risk factors

In this study, potential explanations for the high incidence rate from the medical perspective comprise the early stage of infection of the index person(s) (all participants were asymptomatic upon arrival), the age of the participants (mean age for the respondents was 69.3 years), and the act of singing. He et al. [13] showed that infectiousness was at highest at the presymptomatic stage or at symptom onset, and a meta-analysis of 59 studies revealed that individuals aged over 70 years had 65% elevated risk for COVID-19 [32]. However, since age is a known risk factor for more severe symptoms, sampling error should be kept in mind as in some countries during the pandemic only those with severe symptoms or with direct contact with a SARS-CoV-2 -positive person were tested [33]. The pre-existing comorbidities in our participants may explain the severity of the disease; the two hospitalized individuals had diagnoses of obstructive sleep apnea (OSA) and diabetes multiple diagnoses associated with more severe disease including obstructive sleep apnea (OSA), overweight, hypertension, heart problems and diabetes. In a cohort study of 174 568 adults with SARS-CoV-2, 25.9% of hospitalized patients had diabetes [34], and in several studies OSA has been found to be associated with more severe COVID-19 [20,35,36]. It is also noteworthy that the one singer who was not infected in this event had previously recovered from COVID-19 and also had no underlying diseases.

Factors known to protect an individual from COVID-19 infection include masks or respirators [37], vaccinations [38], and previous COVID-19 infection [39]. At the time of the choir rehearsal here, no vaccines were yet available in Finland. The mask recommendation concerned mainly public transport and transit to tests or quarantine. Masks and respirators are surely uncomfortable when singing, but encourage especially people in risk groups to wear protection. This is in line with e.g., the US Centers for Disease Control and Prevention (CDC) recommendation [40]. Previous COVID-19 infection was observed to be a protecting factor also in our study, as the only non-infected participant had recovered from COVID-19 eight months earlier.

### Limitations of the study

At the time of the studied event, the main variant in Finland was the wild-type SARS-CoV-2, and the first alpha B.1.1.7 variant was detected 5 weeks later [41]. Avoiding gatherings of more than 10 persons was recommended [42]. Also recommended was forgoing hobby activities in groups at the time of the event, but only in situations where close contact or the risk of droplet infection at close range could not be avoided [43]. Altogether there were 1666 recorded COVID-19 cases (qRT-PCR) in the Uusimaa area (n = 1 698 974, performed tests 41 769, test positivity 3.99%) [44]. Thus, it is unlikely that there would have been more than one infected person present with a different exposure background.

One could debate whether the participants were infected during the rehearsal. However, the symptoms of the singers started 2–4 days after rehearsal and they tested qRT-PCR -positive at 3–5 days from the rehearsal. This is in line with earlier findings from a human challenge study [45], where the exact moment of infection exposure was known, and participants were systematically followed regarding viral kinetics; symptoms were reported 2–4 days after inoculation, and viral load was detectable from the nose at 58 h. Thus, it can be concluded that the rehearsal fits the timeline of viral kinetics in serving as the probable incubation event. However, no virus sequence typing was carried out. The limitations of the study include potential infections in the cafe or other settings around the choir rehearsal; nevertheless, the presented modeling suggests that all infections may be attributed to the choir rehearsal. The gathering limit of over 10 persons likely reduced additional interactions significantly.

In addition, we note that the model room was a simplified version of the actual interior. The actual interior spaces are highly complex, with moving persons and varying geometric details. Additionally, the exact location of the index person was not known and was set conservatively to be as far from the other singers as possible. Also, the quanta emission rate of the index person was unknown. Here, the choice *q’* = 1000 1/h indicated high infection risk for all choir attendees. This order of magnitude for *q’* is expected and revealed already by simple calculations based on perfect mixing assumption, room volume, and the Wells Riley model.

Ideally, conducting the interviews earlier would have been preferable. However, the necessary process of obtaining research permits in this type of study led to a delay. Despite this, there were no inconsistencies in the interviewees’ recollections, indication accurate recall. Furthermore, patient records were reviewed.

## Conclusions

We demonstrated that airborne transmission is a likely explanatory factor for this mass contagion event. The main numerical finding of the study is that social distancing alone is not an effective mitigation strategy for airborne viruses in indoor spaces. The combination of high-filtration masks and a further focus on indoor air quality may be necessary to avoid superspreading, particularly when the exposure time is long. This study highlights the importance of informing people in risk groups and their close ones to protect themselves during a pandemic.

## Supporting information

S1 Survey Survey questions on choir member characteristics. Questions translated from Finnish.
(DOCX)
