# Supplementary material for: Superspreading of SARS-CoV-2 at a choir rehearsal in Finland—A computational fluid dynamics view on aerosol transmission and patient interviews
Source: PLoS One. 2024 Sep 12;19(9):e0302250. doi: 10.1371/journal.pone.0302250 (PMC11392323; doi:10.1371/journal.pone.0302250)
Supplement: S1 Survey — Questions translated from Finnish. (DOCX) [file pone.0302250.s001.docx]

Supplement 1

Choir member characteristics - survey questions (translated from Finnish)

Choir rehearsal

1. Did you attend the rehearsal 16 November 2020?
2. Did you visit the café afterwards?
3. Did you attend the karaoke afterwards?
4. Where were you seated?
5. Did you wear any PPE (personal protective equipment)?
6. How was the safety distancing applied? Did you follow the 2 meter distancing rule?
7. Did you have any closer contact (i.e. hand shaking or hugging) to other choir members?

At the time of the rehearsal:

1. Height, weight, age, gender?
2. Diagnoses and medications?
3. Covid vaccination?
4. Smoking status

Possible COVID-19 symptoms and diagnosis:

1. Previous COVID-19 infections before the rehearsal?
2. Did you have any symptoms related to infection at the time of the rehearsal?
3. Date for first symptoms after the rehearsal?
4. Please describe all the symptoms
5. Reverse transcription polymerase chain reaction (qRT-PCR) test date and result?
6. Were you hospitalized? If yes, when, where, why and for how long?
